# Microsecond MD simulations of human CYP2D6 wild-type and five allelic variants reveal mechanistic insights on the function

**DOI:** 10.1371/journal.pone.0202534

**Published:** 2018-08-22

**Authors:** Charleen G. Don, Martin Smieško

**Affiliations:** Molecular Modeling Group, Department of Pharmaceutical Sciences, University of Basel, Basel, Switzerland; Faculdade de Ciências da Universidade de Lisboa, PORTUGAL

## Abstract

Characterization of cytochrome P450 2D6 (CYP2D6) and the impact of the major identified allelic variants on the activity of one of the most dominating drug-metabolising enzymes is essential to increase drug safety and avoid adverse reactions. Microsecond molecular dynamics simulations have been performed to capture the dynamic signatures of this complex enzyme and five allelic variants with diverse enzymatic activity. In addition to the apo simulations, three substrates (bufuralol, veliparib and tamoxifen) and two inhibitors (prinomastat and quinidine) were included to explore their influence on the structure and dynamical features of the enzyme. Our results indicate that the altered enzyme activity can be attributed to changes in the hydrogen bonding network within the active site, and local structural differences in flexibility, position and shape of the binding pocket. In particular, the increased (CYP2D6*53) or the decreased (CYP2D6*17) activity seems to be related to a change in dynamics of mainly the BC loop due to a modified hydrogen bonding network around this region. In addition, the smallest active site volume was found for CYP2D6*4 (no activity). CYP2D6*2 (normal activity) showed no major differences in dynamic behaviour compared to the wild-type.

## Introduction

Genetic polymorphism in CYP2D6, a monooxygenase enzyme metabolizing around 25% of the therapeutic drugs [1], frequently leads to altered enzyme activity (increased, decreased or none) which in turn has an impact on the drug efficacy and the occurrence of adverse reactions [2]. Depending on the genetic variant, four phenotypes can be assigned: (i) ultrarapid metabolizer (UR), (ii) normal metabolizer (NM), (iii) intermediate metabolizer (IM) and (iv) poor metabolizer (PM) [3]. During the last decade, increased awareness concerning the risks that CYP2D6 polymorphism can bear on treatment outcome has lead to relabeling of several CYP2D6 metabolized drugs with additional guidelines on drug dosage in case of polymorphism by the FDA [4]. In addition, the clinical pharmacogenetics implementation consortium (CPIC) has been procuring therapeutic guidelines for several drugs that have a high risk of adverse reactions when administered to patients with a phenotype deviating from the normal CYP2D6 activity (wild-type) [5].

The enzymatic activity can be altered in various ways. Intrinsic properties of CYP2D6 such as a localized hydrophobic region in the binding pocket (V119, F120, L121, F219, L220, R221, V370, P371, L372, V374) or the strong electrostatic field of the two carboxylates (E216 and D301) enabling binding and orienting of the ligand, are essential for its unique substrate specificity (6–8). In addition, the ligand flux between the exterior and the buried active side is tightly regulated through the formation of tunnels [6]. Environmental conditions have been shown to contribute to the metabolic capacity of the enzyme, too. Its redox partner the cytochrome P450 reductase (CPR/POR) provides the electrons needed for the redox reaction [7]. The presence of other isoforms is also important as it has been observed that P450s (including CYP2D6) are able to form homomeric and heteromeric complexes [8]. In addition, the polarity and organization of the membrane is essential for proper anchoring and enzymatic function [9].

How all these factors exactly influence and modify CYP2D6 function on the molecular level is still poorly understood.

Using *in silico* methods for extending our understanding of the enzyme’s essential bond-forming capabilities, channel formation and overall plasticity is burgeoning [10–17]. This trend is driven by the growing amount of structural data (the majority of available mammalian CYP2D6 x-ray structures have been released after 2014) as well as ever increasing computational power allowing to reach the millisecond time scale of the molecular dynamics (MD) simulations [18]. Such simulations are the method of choice for studying the biomolecular structural and dynamical aspects on atomic level (based on its thermodynamics and kinetics) and conveniently complement experimental investigations [19,20].

In general, MD simulation studies (varying from 5 ns to 250 ns) that focused on CYP2D6 and different variants (CYP2D6*34, CYP2D6*17–2, CYP2D6*17–3, CYP2D6*53, CYP3D6*2, CYP2D6*10, CYP2D6*14A, CYP2D6*51, CYP2D6*62) showed that the global structural fold remains similar for all [12,15,17]. However, local changes mainly found at the loop regions were demonstrated to alter the flexibility (increased/decreased) of one particular variant and they are thought to correlated with the enzyme activity [12,15,21]. Moreover, the CD-, GH-, FG-, and BC loops displayed increased or decreased flexibility compared to the wild-type. Especially the last two loops are positioned close to several important tunnels (2a/2ac/4 and 2b/2e/2c respectively) which allows the regulation of ligand flux between the outer environment and the buried active site. If the loops become more rigid or flexible upon a particular amino acid mutation this will translate into different thermodynamics and altered enzyme activity [17]. It is known that enzymatic reactions occur on the millisecond-to-second time scale, hence the need to prolong the MD simulation studies to a longer time scale in order to capture an improved overall framework of the links between enzyme structure, movement and its catalytic action [22]. Improved information and any novel insights regarding CYP2D6 polymorphs could also potentially contribute to minimizing the interindividual differences in pharmacological and toxicological responses to a drug (e.g. altered binding mode of a drug in the binding pocket of an allelic variant) during drug discovery and translate into more focused pharmacovigilance [23–25].

Our pioneering study focuses on exploring such dynamic phenomena contributing to enzyme activity on a larger time scale (1 μs) for CYP2D6 wild-type and five allelic variants (CYP2D6*2, CYP2D6*10, CYP2D6*17, CYP2D6*4 and CYP2D6*53) (Table 1).

**Table 1:** Overview of all MD simulations performed (1 μs).

Run ID	Description
**wt_a**	CYP2D6*1 (wild-type) apo enzyme (normal activity)
**V2_a**	CYP2D6*2 apo enzyme (normal activity)
**V10_a**	CYP2D6*10 apo enzyme (decreased activity)
**V17_a**	CYP2D6*17 apo enzyme (decreased activity)
**V4_a**	CYP2D6*4 apo enzyme (no activity)
**V53_a**	CYP2D6*53 apo enzyme (increased activity)
**wt_buf**	CYP2D6*1 (wild-type) enzyme run with bufuralol (substrate)
**wt_vel**	CYP2D6*1 (wild-type) enzyme run with veliparib (substrate)
**wt_tam**	CYP2D6*1 (wild-type) enzyme run with tamoxifen (substrate)
**wt_pri**	CYP2D6*1 (wild-type) enzyme run with prinomastat (inhibitor)
**wt_qui**	CYP2D6*1 (wild-type) enzyme run with quinidine (inhibitor)
**V17_qui**	CYP2D6*17 enzyme run with quinidine (inhibitor)
**V17_pri**	CYP2D6*17 enzyme run with prinomastat (inhibitor)
**V53_qui**	CYP2D6*53 enzyme run with quinidine (inhibitor)
**V53_pri**	CYP2D6*53 enzyme run with prinomastat (inhibitor)

**Fig 1:**
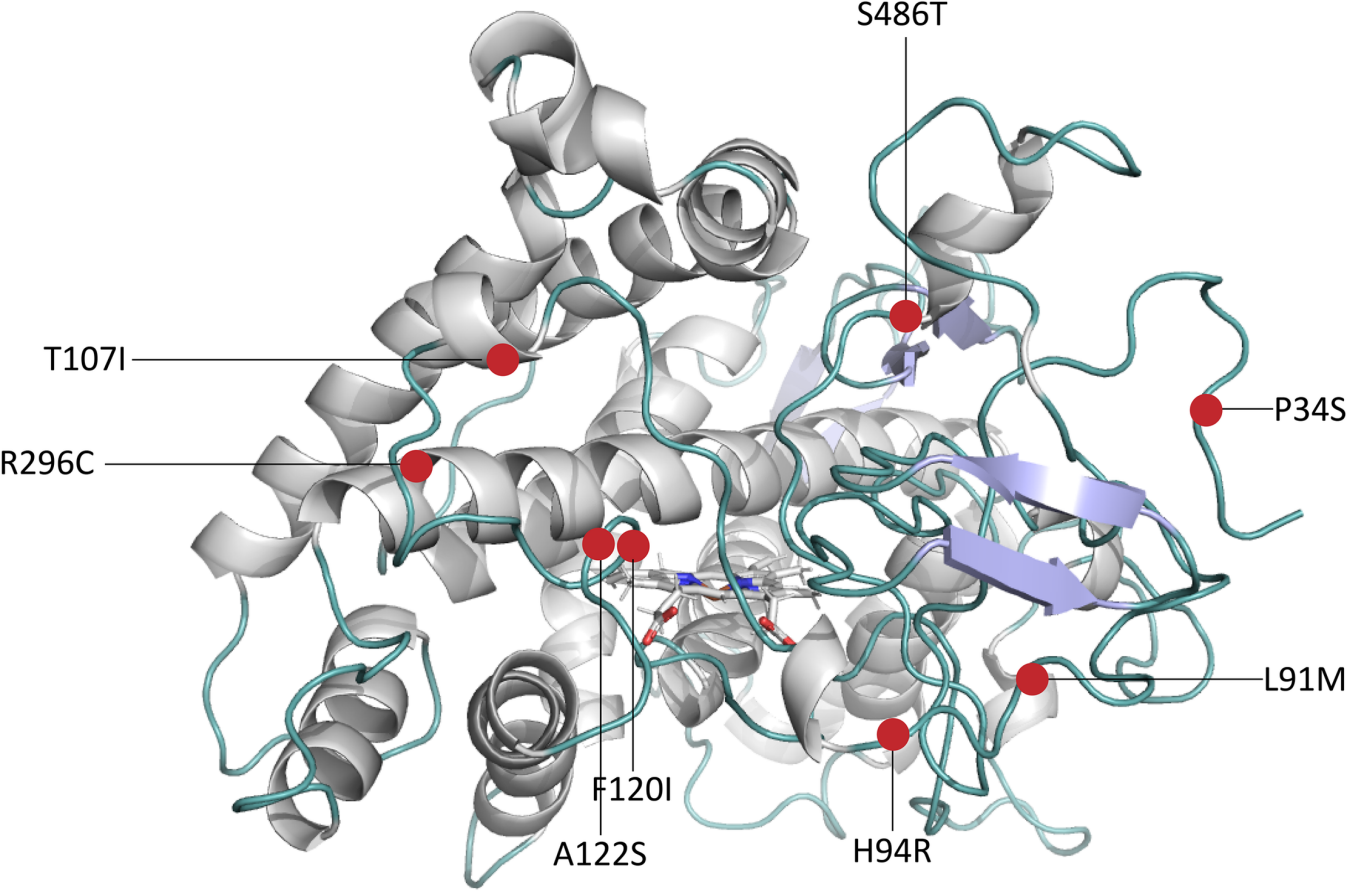
CYP2D6 structure showing the positions of the single nucleotide polymorphisms (SNPs). The SNPs are highlighted in red. SNPs in CYP2D6*2 are R296C and S486T, in CYP2D6*10 are P34S, S486T in CYP2D6*17 are T107I, R296C and S486T, in CYP2D6*4 are P34S, L91M, H94R and S486T, in CYP2D6*53 F120I and A122S (see overview Table 2 and Fig 2).

The selection criteria of the CYP2D6 variants were procured based on (i) the functional activity and clinical relevance, for each phenotype at least one variant was selected (Table 1). At the moment, only one CYP2D6 variant with increased *in vitro* activity is identified. A list containing all currently identified allelic CYP2D6 variants (> 100) can be found at www.pharmvar.org. (ii) the location and overlap of the mutations within the CYP2D6 structure (Fig 1): except for CYP2D6*53 (increased activity) all the other selected variants have at least one mutation in common. Furthermore, three substrates and two inhibitors were selected in order to investigate the way they are accommodated in the active site and influence the enzyme flexibility.

**Table 2:** Overview of the CYP2D6 allelic variants together with information regarding their relevance and impact on the enzyme.

CYP2D6 Allele	Sequence modification ^A^	CYP2D6 activity	Nearest SRS^B^	Allelic frequency [34] (%)^C^:
**Normal Metabolizer (NM)**				
*1 (wild-type)	None	Normal		Americans (50%), Caucasians (38%)
*2	R296C, S486T	Normal	6	Asians (central, 30%), Caucasians (4%)
**Intermediate Metabolizer (IM)**				
*10	P34S, S486T	Decreased	5/6	Asians (east, 43%), Caucasians (2%)
*17	T107I, R296C, S486T	Decreased	1/3/4/6	Africans (19%), Caucasians (0.3%)
**Poor Metabolizer (PM)**				
*4	P34S, L91M, H94R, S486T	Inactive	5/6	Caucasians (18%)
**Ultrarapid Metabolizer (UM)**				
*53	F120I, A122S	Increased	1	*Global frequency data incomplete*

^A^ data obtained www.pharmvar.org accessed on 20.12.2017

^B^ Substrate Recognition Site (SRS)

^c^ the population identified to have the highest occurrence regarding the CYP2D6 allele is indicated first, followed by the occurrence found for Caucasians.

The 15 MD simulations performed in this study extend our existing knowledge on the structural and functional relationship of CYP2D6 wild-type and five variants. Our results suggest that the altered enzyme activity can be attributed to both changes in hydrogen bonding network within the active site as well as local structural differences in flexibility, position and shape of the binding pocket–in particular the loop regions (FG and BC) essential for the regulation of the ligand access to the heme.

### Single nucleotide polymorphisms (SNPs) of the five CYP2D6 variants

**Fig 2:**
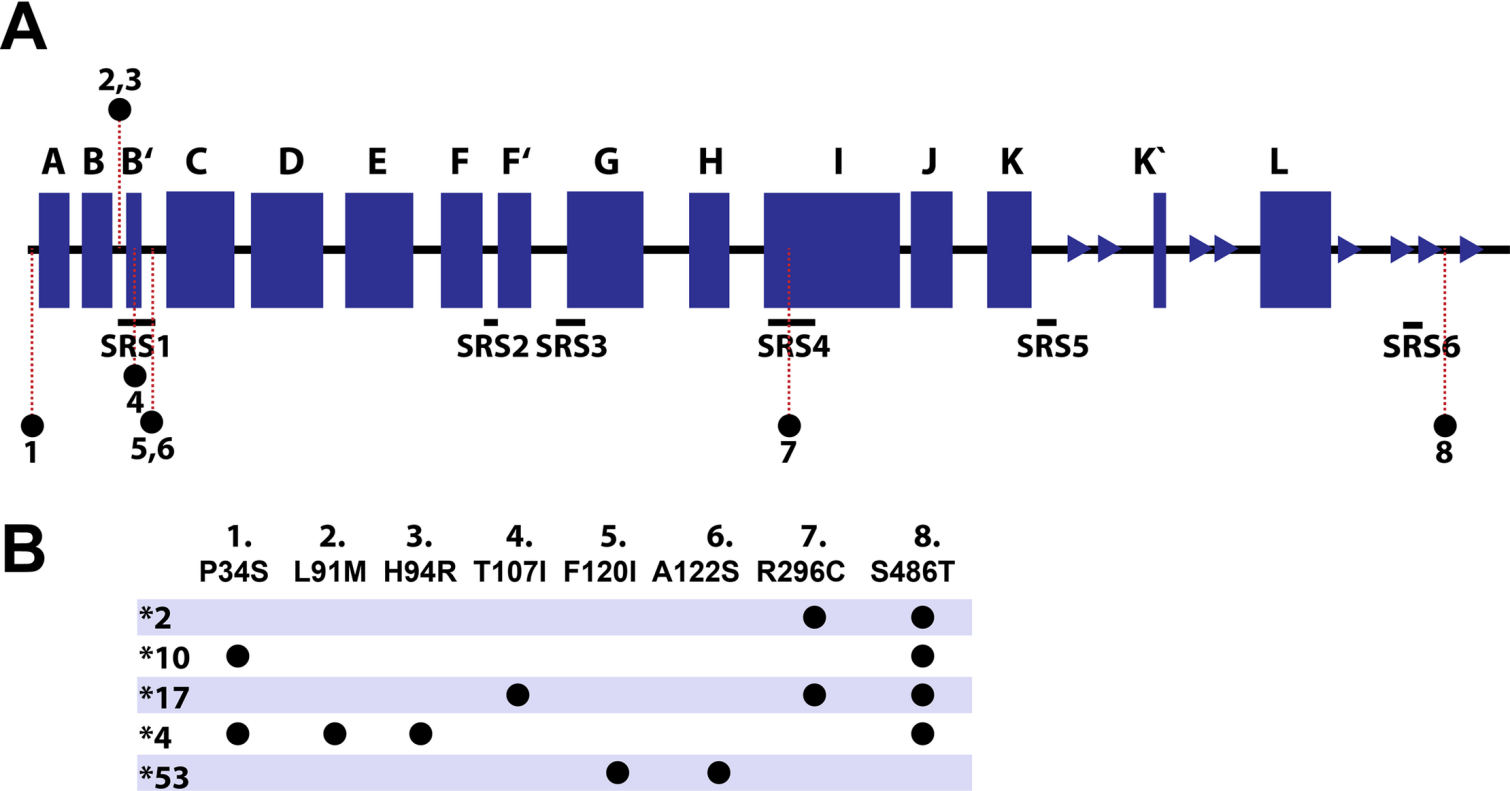
Overview of the CYP2D6 sequence, the secondary elements and the amino acid mutations for the five variants. (**A**) The CYP2D6 sequence is shown together with the location of the substrate recognition site (SRS1-SRS6) and the location of the mutations. (**B**) The table displays the CYP2D6 allelic variants and their mutations.

Binding specificity and enzyme activity is controlled by a diversity of factors as mentioned in the introduction. The amino acid constitution of the enzyme contributes largely to its stability (e.g. intra-molecular hydrogen bonding network) and function (e.g. more hydrophobic lining channel residues regulating typical lipophilic substrate flux to and from the heme) [1]. Depending on the location of mutation (e.g. surface, substrate recognition site or active site) and its nature (e.g. hydrophobic into hydrophilic) the impact on the enzyme activity and stability will be more or less pronounced. For the eight SNPs among the CYP2D6 variants in this study (Figs 1 and 2), half of them caused a polarity change and three reversed hydrophobicity or hydrophilicity (S1 Table).

The SNP P34S in CYP2D6*4 and CYP2D6*10 is known to perturb the proline-rich PPGP motif near the N-terminus site crucial for proper folding and membrane anchoring of the enzyme [26]. It has been observed that P34S is solely responsible for decreased to almost abolished enzymatic activity [27]. Both F120I and A122S SNPs in CYP2D6*53 (increased activity) are located at the BC-loop, in close vicinity of the CYP2D6 iron-heme. Several site-directed—and molecular modeling studies have proven the relevance of Phe^120^ to CYP2D6 substrate binding, orientation and regiospecificity of CYP2D6 [28–30]. Substitution of Phe^120^ by Ile is expected to reduce the local stacking interactions (increase BC loop flexibility) and to give substrates easier access towards the iron-heme. CYP2D6*2 with R296C located at the I-helix (N-terminus side) and S486T located at the β 4–2 loop, has a similar activity compared to the wild-type [31]. Both the more conserved Ser^486^ substitution with Thr and positively charged Arg^296^ substitution with Cys seem not to have a major impact on CYP2D6 [17,32]. These two SNPs are also found in CYP2D6*17 (decreased activity) in addition to the SNP T107I. The significant role of the latter residue has been demonstrated by experimental research in which decreased enzymatic activity was observed with only the SNP T107I [33]. The hydrogen bond forming residue Thr^107^ is located at the B’ helix in the center of the BC-loop. It can be assumed that substitution with Ile will increase local hydrophobicity and interactions, which in turn will stabilize the structure and reduce the flexibility of the BC loop, potentially resulting in a decreased enzyme activity.

Among Caucasians, the average identified allelic frequencies are 38%, 18%, 4%, 2%, 0.3%, for the wild-type (CYP2D6*1), CYP2D6*4, CYP2D6*2, CYP2D6*10, and CYP2D6*17 respectively [34]. The global distribution regarding the allelic frequency of CYP2D6*53 is not yet available.

## Materials and methods

### *In silico* approach

#### PDB selection

**Fig 3:**
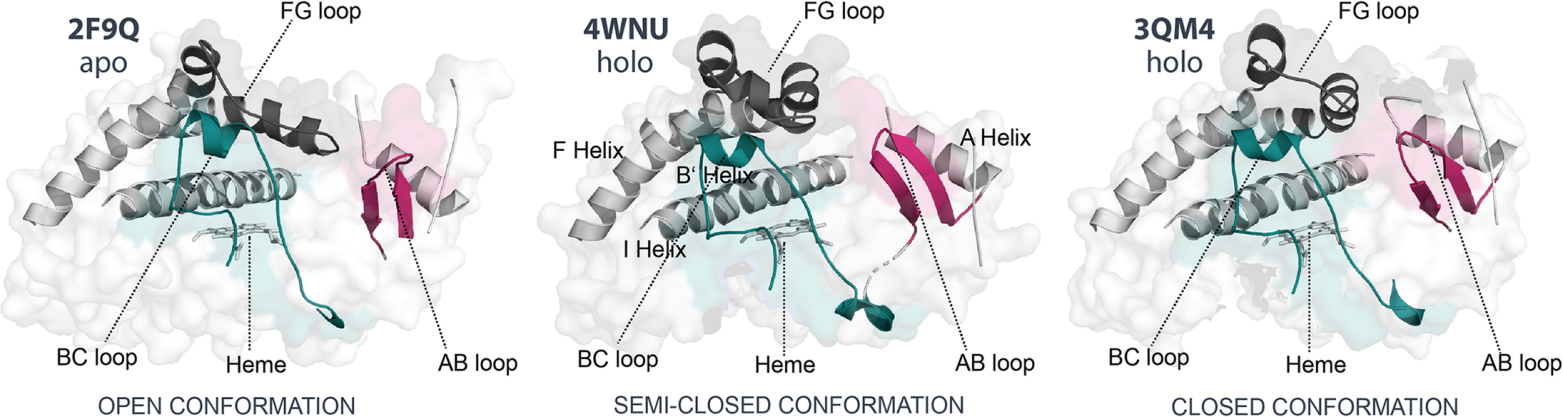
CYP2D6 x-ray structures illustrating the conformational diversity (open/closed). The apo structure 2F9Q (left) shows a more open conformation as defined by the distances between the FG- (grey), BC- (cyan) and AB- (pink) loops. The holo 3QM4 complex (with prinomastat, right) represents one of the most closed CYP2D6 conformations, whereas the holo 4WNU complex (with quinidine, middle) a semi-closed conformation.

It has been observed that the presence of a ligand in the binding pocket induces conformational changes mainly at helices A, B, F, G, the first β-sheet, and loops BC, AB, and FG. The distance between the later three loops and the position of their connecting helices determine a more closed/open protein conformation (Fig 3) [21,35]. A start conformation with an overall fold that is semi-closed was preferred for this study and the CYP2D6 quinidine complex 4WNU was selected [36].

As with all available mammalian CYP2D6 x-ray structures, part of the N-terminus (till G31) was truncated to increase its solubility and facilitate the crystal growing process. Furthermore, residues 38–52, 145–147 and 498–501 all located outside the active side, were missing in chain A. In our model the missing residues were filled in using Prime [37]. The iron-heme was modelled as Fe^3+^. This oxidation state corresponds with the active catalytic ferrous state [38].The Schrodinger Protein Preparation Wizard was used for pre-processing of the x-ray structure [39]. Cofactors (5 molecules) that were within the binding pocket were replaced (at their oxygen atom positions) with water molecules: glycerol (replaced with 3 waters) and DMSO (replaced with 2 waters). To optimize the geometry of residues P41 and G42 in the x-ray structure, an additional loop refinement for residues 31–58 was performed using Prime. The resulting structure was minimized using a hybrid method of the steepest decent and the limited memory Broyden-Fletcher-Goldfarb-Shanno (LBFGS) algorithms with a convergence threshold of 0.3 kcal mol^-1^ Å^-1^. Subsequently, this structure was used for generation of all the CYP2D6 variants. After the applied mutations to the structure, each new protein structure was minimized again with the same parameter settings as for the wild-type.

#### Ligands

**Fig 4:**
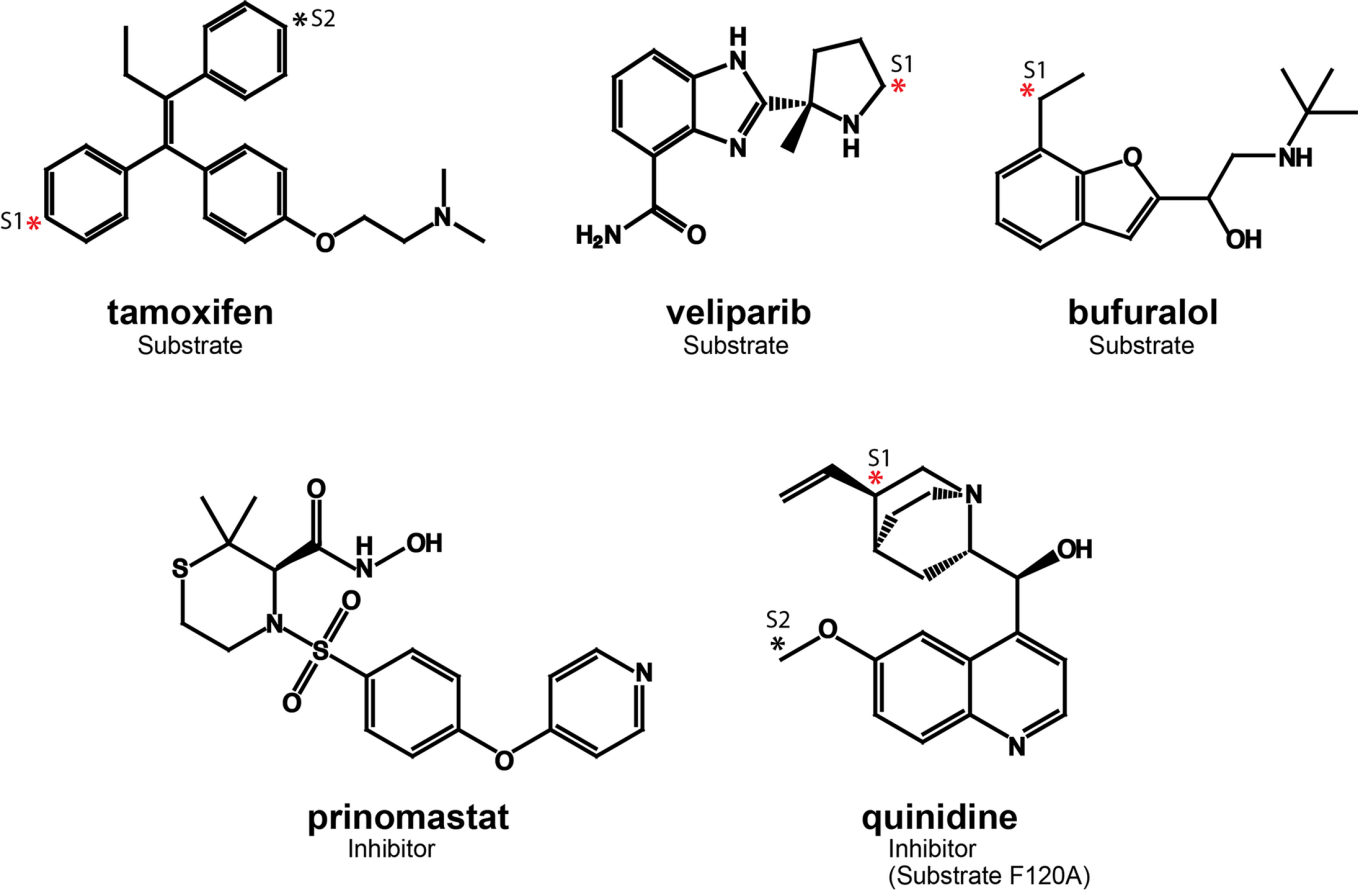
Overview of the CYP2D6 ligands. Three substrates (tamoxifen, veliparib, and bufuralol) and two inhibitors (prinomastat and quinidine) were used in this study. The red asterisk indicates the primary site of metabolism (SoM, S4 Fig), and the black asterisk a secondary SoM. Quinidine might be metabolized in CYP2D6*53, hence the known SoMs are indicated as well.

The ligand selection was based on several criteria: (i) classical CYP2D6 binder; (ii) if there was data available for the ligand from previous computational/experimental studies; (iii) clinical relevance (Fig 4). Bufuralol (β-blocker) and quinidine (antiarrhythmic drug) were selected as classical CYP2D6 substrate and inhibitor respectively. Next, substrates, tamoxifen (estrogen receptor antagonist) and veliparib (PARP inhibitor) were included, as any novel (polymorphism) information regarding their binding behaviour would be relevant from a clinical perspective and potentially contribute to the future of personalized medicine/anti-cancer drug development. In addition, for quinidine and prinomastat (matrix metalloproteinase inhibitor) a crystal structure is available which allows comparing of the dynamical and docking simulations with their observed native pose (S3 Table) [40].

CYP2D6 metabolizes bufuralol into 1’-hydroxybufuralol [41], tamoxifen into 4-hydroxyltamoxifen [42], and veliparib into a lactam containing metabolite [43]. Although quinidine contains the usual CYP2D6 substrate characteristics (basic nitrogen, and flat hydrophobic moiety) it is first metabolized into 3-hydroxy- and O-demethylated quinidine when Phe^120^ is mutated to alanine (or also by E216Q/D301Q) [1,44]. Therefore, the F120I mutation can be expected to allow quinidine to be metabolized. The sites of metabolism (SoMs) for studied ligands are indicated in Fig 4 and their distance to the heme in Supplementary S5 Fig.

As a note, the enzyme kinetics is a complex problematic where one needs to consider numerous possibilities on how a molecule can bind the enzyme. For example, the metabolite of a substrate can act as an inhibitor or, if a substrate has a slow off-rate, one could argue, if it thereby also acts as inhibitor. However, these effects are outside of the scope of this study. A detailed discussion on enzyme binding, the associated terms and kinetics can be found in the literature (41,42).

The binding pose of the ligands tamoxifen, veliparib and bufuralol within the CYP2D6 pocket used for MD simulations was generated using our in-house docking software (DOLINA) [45]. The generation of the ligand poses is based on pharmacophore matching and allows for local induced-fit changes by combinatorial rearrangement of the binding site side chains. The highest ranked pose was selected as a start conformation for MD simulations. For both prinomastat and quinidine the conformation was adapted from the corresponding CYP2D6 x-ray complex (3QM4, 4WNU).

#### Molecular dynamics simulations

Each prepared CYP2D6 variant was solvated using TIP3P water model in an orthorhombic box with 10.0 Å cut-offs from the protein in each dimension, and the net system charge was neutralized by adding counterions (sodium ions). The OPLS_2005 force field as implemented in Desmond (version 2016–4) was used [46]. The system was minimized using a steepest decent algorithm until a gradient threshold of 0.1 kcal mol^-1^ Å^-1^. The dimension of the box for each prepared CYP2D6 variant system (after minimization) were on average 86 x 82 x 87 Å^3^. The production simulations with the total duration of 1.0 μs (NPT ensemble and standard conditions T = 300 K, p = 101.325 kPa) and with frames sampled every 100 ps (in total 10 000 frames were saved per simulation) were performed. The simulations were performed under NPT ensemble, and the Nose-Hoover thermostat at a relaxation time of 1.0 ps with the Martyna-Tobias-Klein barostat were combined with a relaxation time of 2.0 ps at 300 K. The Particle Mesh Ewald (PME) method was used to treat the long-range interactions, the cutoff for short-range interactions was set to 9 Å. Bonds to hydrogen atoms were constrained with the M-SHAKE algorithm and no hydrogen mass partitioning was applied. The two replica simulations were started with different initial velocities. On average one microsecond simulation took 10 days to finish (61195 atoms) on one GeForce GTX Titan GPU.

## Results and discussion

### Overall fold and system equilibration

**Fig 5:**
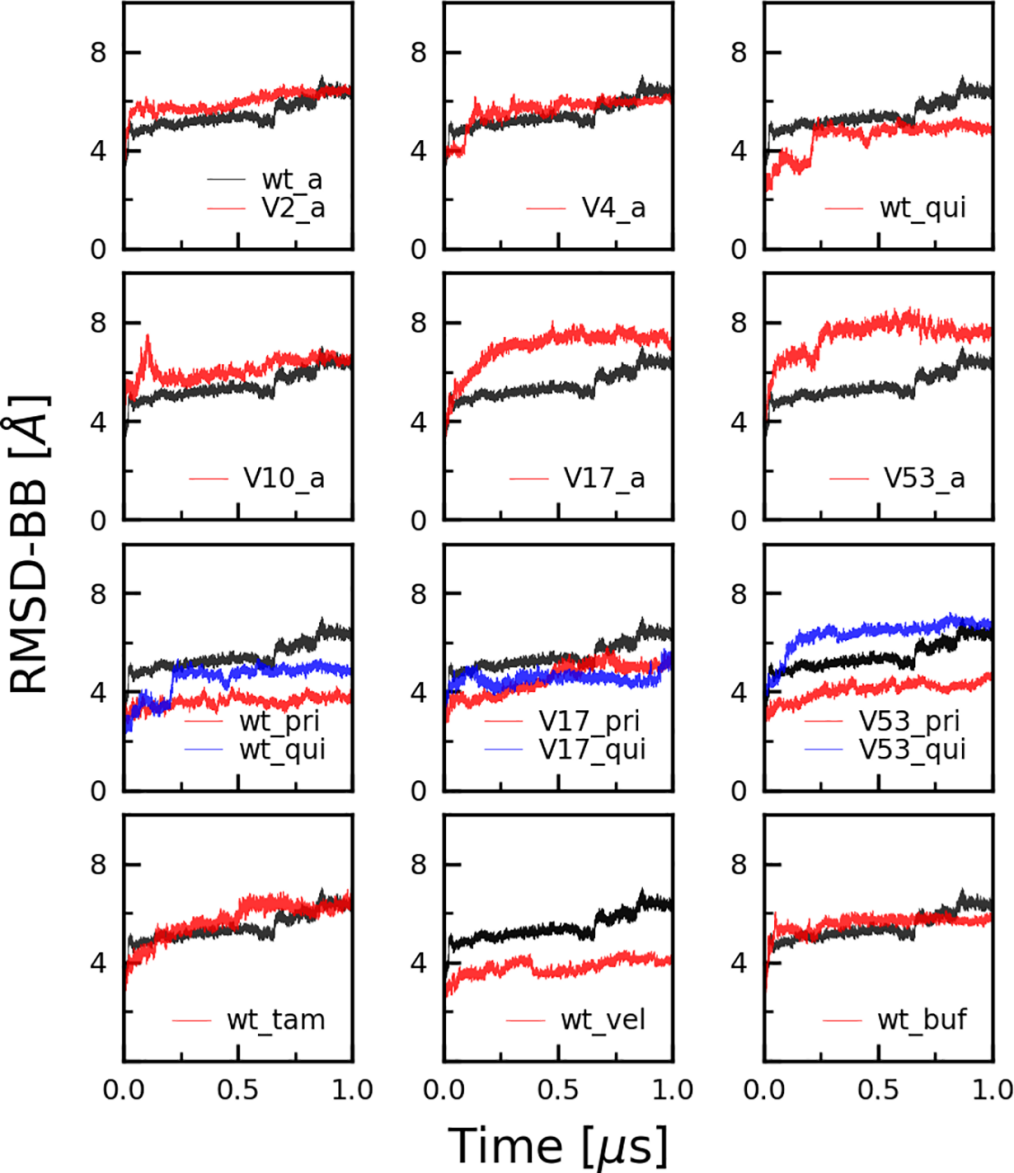
Backbone root mean square deviation (RMSD) graphs for all CYP2D6 simulations. The wild-type simulation is shown black in all graphs. The convergence time varied between 20 ns ≤ t ≤ 250 ns. Compared to the wild-type apo simulations; low degree of fluctuation was observed for wild-type veliparib (wt_vel), quinidine (wt_qui), and prinomastat (wt_pri) and the most flexible variant was CYP2D6*53 apo (V53_a) followed by CYP2D6*17 apo (V17_a).

The root mean square deviation (RMSD) measured over the whole trajectory (1 μs) shows that the wild-type (CYP2D6*1) and all other CYP2D6 variant simulations reached equilibrium at or below the RMSD of 6 Å from the starting structure (Fig 5). S2 Fig displays only the last 100 ns and S3 Fig additional 400 ns of simulation time (1,4 μs simulation in total, only ran for the wild-type apo structure) confirming that all systems were properly equilibrated. In addition, two replica simulations were performed with the apo wild-type using different starting velocities to assure the integrity of the system (S4 Table). The RMSD and RMSF values of the two replica were calculated and compared to the wild-type showing that the intrinsic properties of the system remained conserved in the replicas. Time to convergence varied between 20 ns ≤ t ≤ 250 ns. Fast convergence (t ≤ 50 ns) is observed for most of the wild-type simulations (wt_apo, wt_pri, wt_vel, wt_buf) as well as for CYP2D6*2 (V2_a). The dynamic global and local fluctuations of CYP2D6*2 (mutations R296C and S486T) are expected to be similar to the wild-type as they share the same enzyme activity. All other apo CYP2D6 variants (V10_a, V17_a, V4_a and V53_a) needed longer time (100 ns ≤ t ≤ 250 ns) before reaching equilibrium. This is likely related to the mutations applied, causing local structural instabilities requiring longer times to converge (equilibrate). In general, simulations run with a ligand reached plateau in a short time (t ≤ 50 ns). Wild-type simulations run with quinidine (wt_qui) and tamoxifen (wt_tam) needed longer time (100 ns and 250 ns respectively) to reach equilibrium.

From the apo state simulations, CYP2D6*53 showed the largest conformational change followed by CYP2D6*17, whereas CYP2D6*2, CYP2D6*10 and CYP2D6*4 resembled the dynamics of the wild-type. Furthermore, except for CYP2D6*53 with quinidine (V53_qui), the simulations including the inhibitors prinomastat and quinidine (wt_qui, wt_pri, V17_qui, V17_pri and V53_pri), and the substrate veliparib (wt_vel) led to a more consistent protein conformation (RMSD curves 1 Å to 2 Å bellow the wild-type apo). CYP2D6*53 with quinidine (V53_qui) shows more conformational transitions (higher RMSD values) compared to prinomastat (V53_pri). In the CYP2D6*17 simulations, the presence of both inhibitors (V17_qui, V17_pri) showed a similar conformational stability.

**Fig 6:**
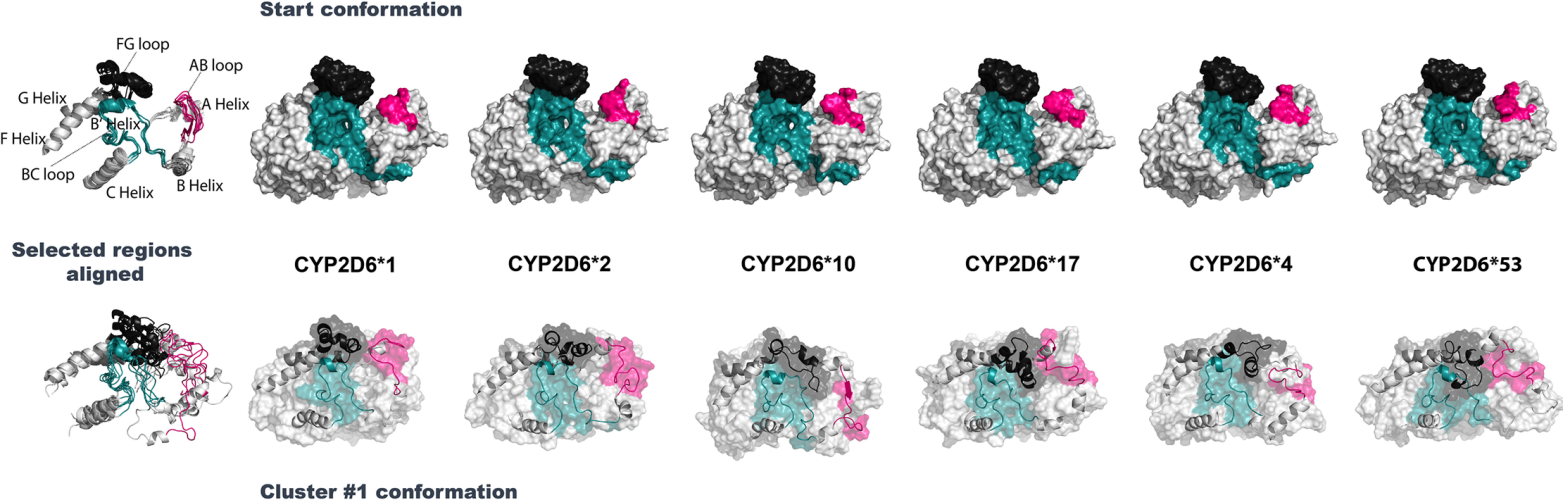
The CYP2D6 starting—and most representative conformation during MD simulations for CYP2D6*1 (wild-type) and the five variants. Structural elements relevant for defining a more open or closed conformation are the regions of the BC loop colored cyan, AB loop colored pink, and FG loop colored black. (**Top**) For all apo CYP2D6 variants, the surface of the start conformations is shown at the beginning (t = 0 μs) of the simulation. All CYP2D6 variants displayed a similar start conformation with an average distance of 13 Å between the FG loop region and AB loop region. (**Bottom**) For all apo CYP2D6 variants, the most populated conformation (obtained by the RMSD clustering) during the simulation are displayed, revealing a semi-closed fold compared to the start conformation. The surface (transparent) is overlaid with the cartoon representation of the selected regions. The alignment in the first image shows that the FG, BC and AB loops are at different positions in the most prevalent conformation for all the CYP2D6 variants.

For all apo simulations the start conformation and the most populated (representative) cluster conformation during the 1 μs trajectory can be found in Fig 6. Overall RMSD values between the CYP2D6 variant apo start conformation and the most populated cluster conformation (cluster #1, occurrence > 60%) calculated for all CYP2D6 variant simulations (backbone carbon atom alignment) varied between 2.2 Å and 3.3 Å. CYP2D6*17 featured the highest ΔRMSD (3.3 Å) whereas CYP2D6*17 with quinidine the lowest (2.2 Å) ΔRMSD was seen. Though the start conformations are very similar to each other (ΔRMSD < 1.3 Å) the clustered conformations are locally more diverse and display a semi-closed fold (ΔRMSD > 3.3 Å).

The active site volume increased upon reaching equilibrium, mainly attributed to a rearrangement of the FG-, BC-, and AB- loops and the connecting helices (F, G, I A, and B) as determined in other studies as well (S2 Table) [14]. Compared to the apo wild-type, CYP2D6*2, CYP2D6*10, CYP2D6*17 and CYP2D6*53 were found to display a more confined fold of the active site, whereas CYP2D6*4 showed a different arrangement mainly of the FG loop and AB loop (more towards the outside) leading to a less confined cavity (more open substrate access state) and a lower volume (8% decrease compared to wild-type apo). The volumes for the wild-type holo conformations varied largely compared to the apo wild-type (from -11% to 26%) suggesting not all ligands possess equal capabilities to favorably engage with the active site and stabilize the protein structure upon binding. The F120I mutation in the active site of CYP2D6*53 contributed to larger observed volumes compared to all others (on average +35%).

Protein structure dynamics (e.g. its folding and overall stability) is known to have an impact on its function [47]. The enzymatic catalysis of CYP2D6 can be indirectly influenced by the distribution of the charged residues at the solvent accessible surface (by for example influencing the overall stability of the protein or the ligand access or egress through long-range electrostatics) and the non-polar solvent accessible surface (also known as buried solvent area, BSA). Therefore, the differences in solvent accessible surface area (SASA) (polar/apolar) and BSA were investigated for the wild-type and allelic variants to explore if there were any patterns observed that could be correlated with the altered activity of the variants (S2 Table).

The ratios within each variant between total surface area, SASA (polar/apolar) and BSA seemed to be consistent within the variants. In general, the SASA was found to vary between -2% and +5%, with one outlier: CYP2D6*53 apo had an increased SASA of 8% compared to the wild-type. An explanation for this larger value might be related to the fact that increased enzyme activity is also achieved by an enlargement of the access tunnel(s) connecting the surface with the active site. At the same time, the catalytic efficiency is retained by optimal alignment of the active site with reduced steric hindrance of F120I, allowing to offer larger active site volumes and a higher through-put of substrates compared to the wild-type. Within the apo variants, only CYP2D6*4 showed a remarkable decrease (13%).

The BSA analysis of the holo conformations showed very large volume changes, varying between -38% (wt_tam) and +70% (V17_pri), which makes it difficult to assign any meaning to the observed BSA values other than that enzyme active site is very plastic and can accommodate various ligand shapes and adjust for an optimal interaction. However, considering the known differences among CYP families in charged surface areas which is linked to the binding of CPR, further studies focused on surface properties might prove valuable for deciphering enzyme activity and allostery [48–50].

### Local flexibility differences

To gain insight in the local structural differences among the CYP2D6 variant simulations, the root mean square fluctuation (RMSF) calculations were performed excluding first 250 ns of the overall trajectory, (the average determined equilibration time). The values were normalized and plotted by subtracting the wild-type simulation values from the CYP2D6 variant simulation. Fluctuations around the N-terminus should be interpreted with caution, since this is the site where CYP2D6 is normally anchored to the membrane which would normally stabilize and reduce the local flexibility. Since the simulations are performed without the membrane, higher RMSF fluctuations are expected for this region.

**Fig 7:**
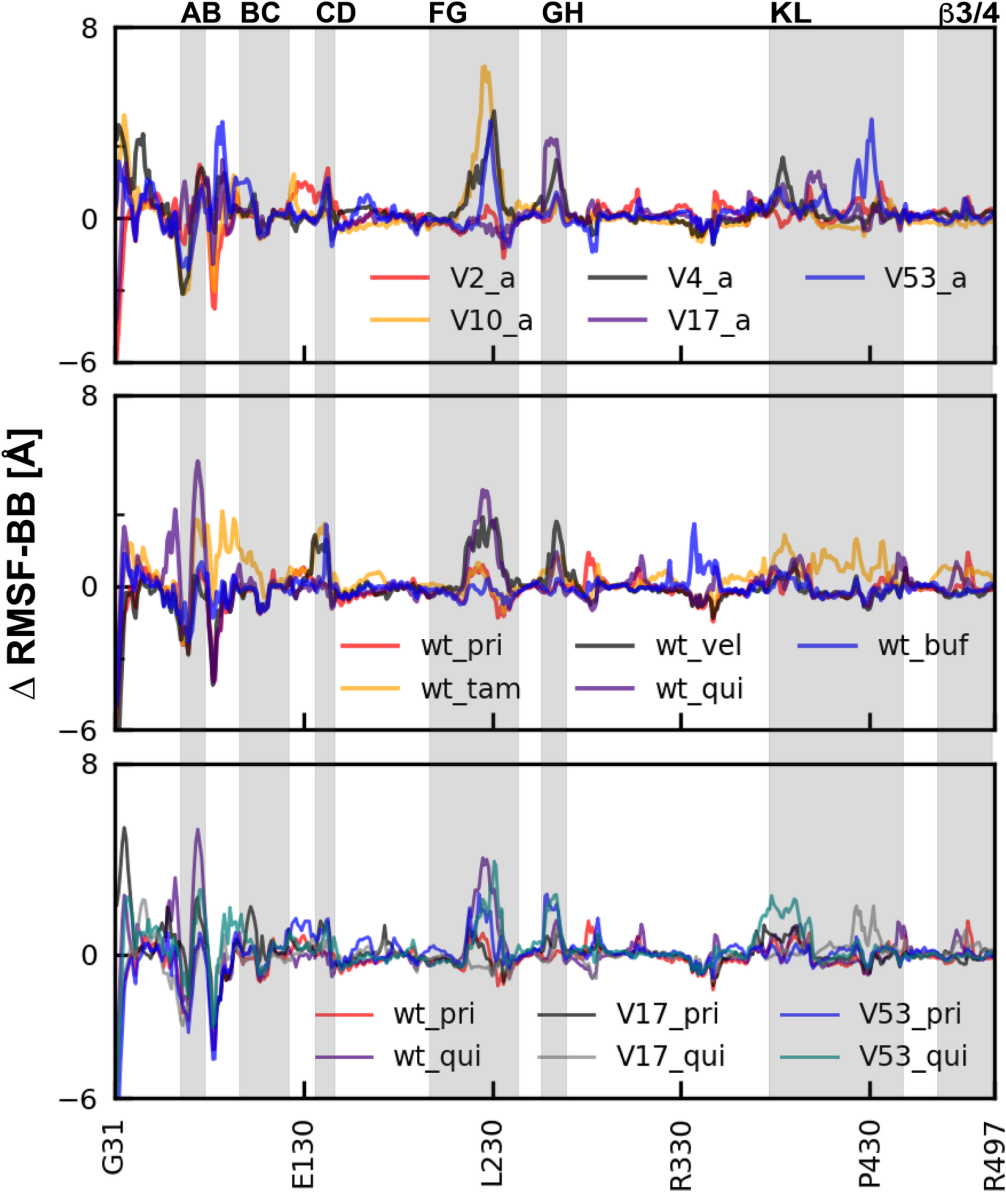
Root mean square fluctuation (RMSF) graphs for all CYP2D6 simulations, excluding the average equilibration time (first 250 ns). In the RMSF graphs, the curves were normalized by subtracting the wild-type RMSF values from the CYP2D6 variant simulation. The grey areas indicate the position of the loop regions. The Largest RMSF fluctuations were found at the B helix and around the loop regions, especially AB, FG, GH and KL loops.

For the apo variant simulations (Fig 7, first row), all loop regions showed larger fluctuations compared to the wild-type, in particular AB, FG, GH and KL loops. In addition, CYP2D6*4, CYP2D6*17 and CYP2D6*53 displayed a larger peak (≥ 4 Å) at the β-2-1, β-2-2 (within the KL loop). CYP2D6*53 displayed the highest flexibility for the KL loop region (P430, E431 and A432). The position of the B helix fluctuated the most for all the CYP2D6 variants, which is likely too high due to the absence of the membrane normally surrounding and stabilizing this site.

Similar flexibility patterns were found for the wild-type simulations ran with ligands (Fig 7, second row). However, the ΔRMSF peaks were in general less intensive which suggests that the presence of a ligand leads to a more stable complex which is in agreement with other studies [12,15]. From all the wild-type holo simulations, quinindine (inhibitor, wt_qui) and veliparib (substrate, qui_vel) induced the largest ΔRMSF peaks (± 3 Å) around the FG and GF loop region, and only bufuralol (wt_buf) showed a pronounced peak (3 Å, D349) located at the J’ helix.

The inhibitor CYP2D6 simulation series (Fig 7, third row) showed similar flexibility RMSF patterns and intensities compared to the wild-type holo simulations. CYP2D6*17 with quinidine (v17_qui) showed a peak close to the N-terminus side of the L-helix, as observed for wild-type with tamoxifen (wt_tam). In addition, CYP2D6*53 with quinidine displayed higher flexibility around the β 2–1, β 2–2 region (within the KL loop).

Considering the important function of the FG- and BC- loop in determining the fold and access towards the iron-heme, in-depth analysis for these two loops were performed separately (S1 Fig) and are commented on bellow.

### Intra-molecular hydrogen bonding network analysis

Hydrogen bonding analysis for the mutated amino acids was performed to deduce any significant structural changes that could affect the enzyme stability for the CYP2D6 variants compared to the wild-type (Table 3). Default Maestro hydrogen bonding criteria were applied (maximal distance from the hydrogen atom to the acceptor atom: 2.8 Å, minimum donor hydrogen bonding angle: 120°, and minimum acceptor Hydrogen bonding angle: 90°).

**Table 3:** Hydrogen bonding network analysis of CYP2D6 variants, focus on mutated amino acids.

CYP2D6 Variant activity (mutations)	Interaction(s) Occurrence (%)			CYP2D6 Variant activity (mutations)	Interaction(s) Occurrence (%)		
CYP2D6*2 similar **(R296C, S486T)**				CYP2D6*10 decreased **(P34S, S486T)**			
wt	R296	+ D252	65%	wt	P34	+ none	
		+ A300	71%				
CYP2D6*2	C296	+ A300	93%	CYP2D6*10	S34	+ none	
wt	S486	+ V480	100%	wt	S486	+ V480	100%
		+ L484	29%			+ L484	29%
CYP2D6*2	T486	+ V480	100%	CYP2D6*10	T486	+ V480	100%
						+ I312	62%
CYP2D6*4 none **(P34S, L91R, H94R, S486T)**				CYP2D6*17 decreased **(T107I, R296C, S486T)**			
wt	P34	+ none		wt	T107	+ N255	58%
CYP2D6*4	S34	+ G36	31%	CYP2D6*17	I107	+ F122	86%
		+ W75	27%			+ L110	38%
wt	L91	+ R88	36%	wt	R296	+ D252	65%
						+ A300	71%
CYP2D6*4	M91	+V87	74%	CYYP2D6*17	C296	+ A300	83%
wt	H94	+ G44	34%	wt	S486	+ V480	100%
		+ R440	72%			+ L484	29%
CYP2D6*4	R94	+ A90	30%	CYP2D6*17	T486	+ V480	100%
		+ E383	33%				
wt	S486	+ V480	100%				
		+ L484	29%				
CYP2D6*4	T486	+ V480	100%				
CYP2D6*53 increased **(F120I, A122S)**							
wt	F120	+ R101	92%				
		+ D301	100%				
CYP2D6*53	I120	+ D301	98%				
wt	A122	+ R441	100%				
CYP2D6*53	S122	+ none					

For each variant the hydrogen bonding partners of the mutated residues were identified and compared with the wild-type. The two SNPs found in CYP2D6*2 (R296C, S486T) showed no significant impact on the hydrogen bonding interactions. The two major backbone–backbone (BB–BB) interactions observed most of the time (> 70%) for the wild-type (Arg^296^ with Ala^300^ and Ser^486^ with Val^480^) were also observed for CYP2D6*2. Indeed, Cys^296^ with Ala^300^ and Thr^486^ with Val^480^ were also identified for CYP2D6*17 (R296C, S486T, T107I) the majority of the time (> 70%). Our observation is that the latter interaction facilitates in stabilization of the β-sheet (β 4–1, β 4–2). The wild-type hydrogen bonding interaction of Thr^107^ with Asn^255^ (G-helix, side-chain(SC)-SC, 58%) was altered for CYP2D6*17. The mutated Ile^107^, located at the BC loop, interacted most of the time with Phe^112^ (86%) and for a smaller time period with Leu^110^ (38%). This could be an indication that in this variant the BC loop is less flexible due to the extended hydrophobic network compared to the wild-type and indirectly contributes to a more flexible FG loop due to the missing N255 bond (as supported by the FG–and BC loop RMSD analysis, see S1 Fig). Based on this data, we propose that the substrate access through the closest tunnels (2c/2e) is hindered in this variant, which might translate to the decreased activity of the enzyme.

Handa et al. performed similar analysis for wild-type and CYP2D6*17, though only over a 5 ns MD trajectory [17]. For the T107I mutation different hydrogen bonding interactions were observed for the wild-type (L110, G11, F112 and V104) and CYP2D6*17 (G111 and F112). For the R296C mutation the wild-type interacted with D252 which is in agreement with our observation, though the A300 interaction is missing in both cases. S486T formed for both wild-type and CYP2D6*17 a hydrogen bond with V480 as seen in our simulations. The difference in results can be attributed to a rather short simulation time of the previous modeling study.

The decreased activity of CYP2D6*10 (P34S, S486T) is mainly caused by the SNP P34S as explained in our SNP section. Though as in the wild-type no hydrogen bonding interactions were observed, it is known that this proline-rich motif (PPGP) is crucial for protein folding [26]. In addition, the hydrogen bonding results for this residue should be treated with caution since our simulations lack the membrane, which would normally stabilize the N-terminal part of the enzyme (including Pro^34^). This is also valid for the P34S mutation found in CYP2D6*4 (P34S, L91M, H94R, S486T).

An interesting observation for CYP2D6*4 was found for the SNP H94R located at the BC loop. Earlier studies indicate that a conserved arginine found in P450s is acting as a gatekeeper of the water tunnel; if interacting with the heme propionates, water molecules are prevented from accessing the active site from the surface [51,52]. Proper regulation of water molecules supply at the active site is crucial for efficient enzyme functioning. In CYP2D6 there are two conserved arginines (R440, R441). In the wild-type His^94^ interacts the majority of the time with Arg^440^ (72%, BB-SC), whereas the mutated Arg^94^ interacted with Glu^383^ and Ala^90^ for around 30% of the time. No compensating hydrogen bonding partners were identified for Arg^440^ in this variant, meaning the side-chain is free to move around and might thereby interfere with the normal functioning of this water channel. Another MD simulation study of 250 ns duration suggested that the hydrogen bonding interactions of the protein with the heme also largely contribute to proper embedding of the heme group within the enzyme [12]. The loss of interactions between the heme and the enzyme (including Arg^441^) likely worsens the heme recognition and incorporation which in turn might lead to no enzyme activity as observed by experiment for CYP2D6*62 [53]. However, analysis of Arg^441^ of both wild-type and CYP2D6*4 showed similar hydrogen bonding interaction profiles (with V119, A122, and the heme porphyrin ring of the heme, > 60%). In addition, no weakened interactions between the heme and the protein were found for CYP2D6*4 and the wild-type.

Therefore, we suggest that the absence of activity for this variant could be related to both (i) the the disruption of the proline rich motif by the SNP P34S preventing optimal enzyme folding and interaction with the membrane, and in addition (ii) the SNP H94R might decrease efficient regulation of the water tunnel by its large positively charged side-chain in such a way hat it decreases enzyme activity.

For the SNP A122S in CYP2D6*53 (F120I, A122S) the Ala^122^ interaction with Arg^441^ found in the wild-type (100%) was lacking for Ser^122^. No compensating protein hydrogen bonding interactions were identified for Arg^441^. Phe^120^ interacted with Asp^301^ and Arg^101^ in the wild-type (> 92%). Though the latter was lacking for Ile^120^, the more important interaction with Asp^301^, known to be essential for the specificity of CYP2D6, was maintained [54]. RMSD analysis of the BC loop showed a 1.5 Å increase in flexibility on average for the BC loop compared to the wild-type (S1 Fig). We would therefore propose that the increase in enzyme activity for CYP2D6*53 is potentially related to the loss of the otherwise BC-loop-stabilizing hydrogen bonding interactions observed in the wild-type.

### CYP2D6 ligand analysis

RMSD calculations were performed for each ligand with the starting coordinates being used as the reference. Most stable dynamics were found for the wild-type with veliparib, quinidine and bufuralol (wt_vel, wt_qui, wt_buf) (S4 Fig). Prinomastat showed the lowest stability among all wild-type holo simulations. Comparison of variants CYP2D6*17 and CYP2D6*53 revealed that the latter induced the largest change from the initial conformation (V53_pri, V53_qui). The spike for prinomastat in CYP2D6*53 around 0.3 μs is due to a shift of the pyridyl ring, which later on is followed by a movement (after 0.5 μs) of the inhibitor closer towards the heme (± 6 Å). In the wild-type and CYP2D6*17 this displacement of prinomastat did not occur. Instead, during the whole simulation time it remained close to the initial pose with small fluctuations of the pyridyl ring. Despite the reduced steric hindrance by the F120I substitution, quinidine was not found to move closer towards the heme during simulation with CYP2D6*53. In all three simulations (wt_qui, V53_qui, V17_qui) it kept its position constant. In the following sections, the functional groups or atoms described in the parenthesis always refer to the ligand.

#### Prinomastat

**Fig 8:**
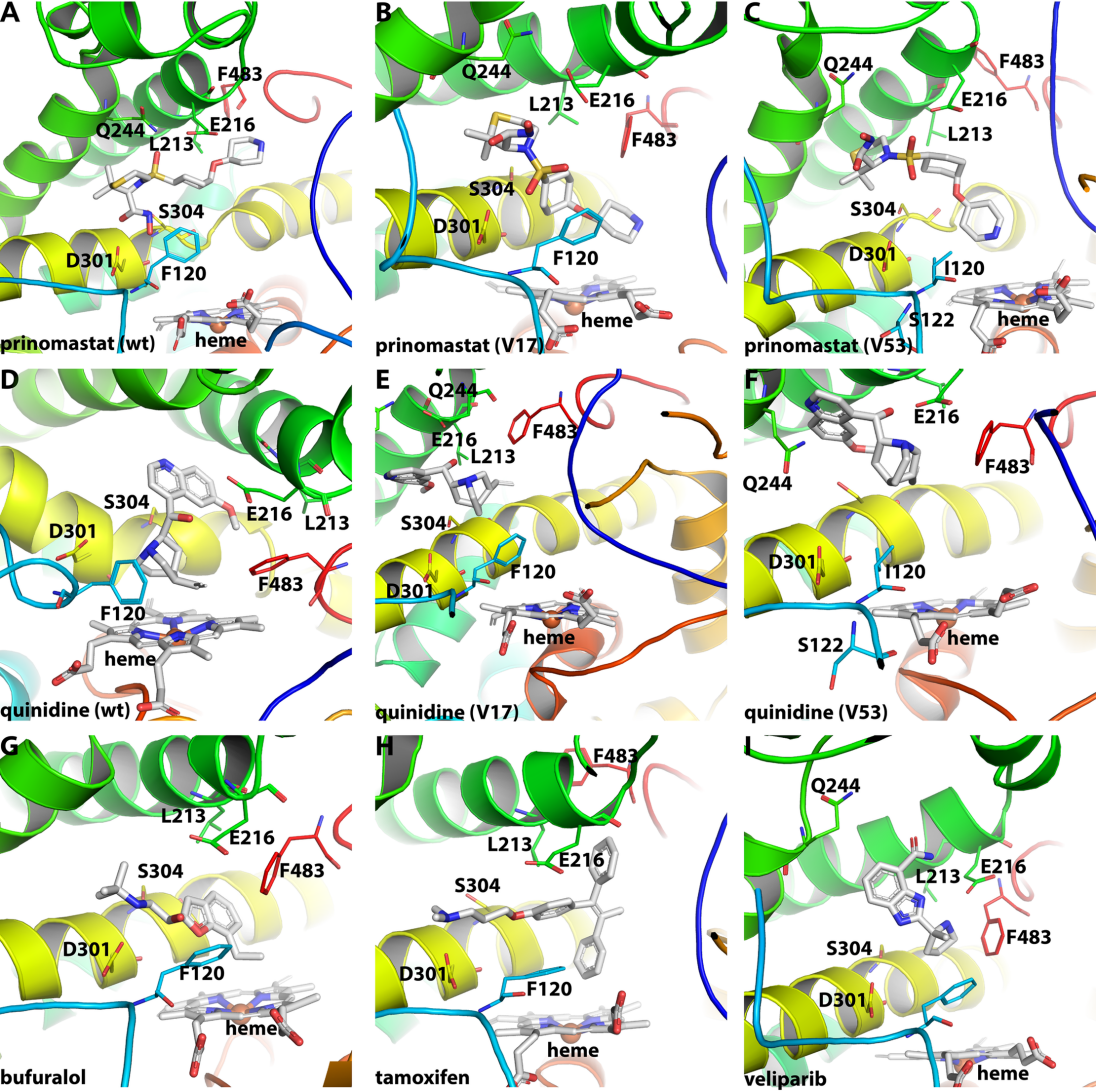
Dominant ligand conformations during 1 𝛍s MD simulation. **(A-C)** Prinomastat (inhibitor) binding mode is displayed for wild-type (A), CYP2D6*17 (B) and CYP2D6*53 (C). **(D-F)** quinidine (inhibitor) binding mode is displayed for wild-type (D), CYP2D6*17 (E) and CYP2D6*53 (F). **(G-I)** The wild-type binding mode is shown for the substrates bufuralol (G), tamoxifen (H) and veliparib (I).

The time-averaged binding mode of prinomastat (inhibitor) in a similar binding mode as observed in the x-ray structure for the wild-type, CYP2D6*17 (3.6 Å) and CYP2D6*53 (Fig 8A–8C). Dominant hydrogen bonding interactions with D301 (hydroxyl group of the hydroxamic acid), E216 and Q244 (with one of the sulfonyl oxygens) were most of the time (> 70%) present in all three cases (wt_pri, V17_pri, V53_pri). Other hydrogen bonding interactions observed for the wild-type for a shorter period of time (25%-50%) included G212, S217, I369, and A482. For CYP2D6*17 and CYP2D6*53 an additional hydrogen bonding interaction was observed with R221 (with oxygen of the hydroxamic acid). During the simulation, the pyridyl nitrogen which is known to be coordinated towards the heme iron for its inhibitor effect [55], was found more often for CYP2D6*17 and CYP2D6*53 in close vicinity (6 Å or 7 Å on average) of the heme than for the wild-type (11 Å on average). Stabilizing hydrophobic interactions included L110, F112, F120, L213, F247, A300, V308, F483, and L484 observed during all three simulations.

#### Quinidine

The quinuclidine moiety of quinidine was pointing in all three cases (wt, V17, V53) towards the heme and the quinolone ring into the direction of the F- and G helices (Fig 8D–8F). Hydrogen bonding interactions were observed in all three cases with E216 (with the protonated nitrogen of the quinuclidine moiety and the hydroxyl hydrogen), Q244 (with the hydroxyl oxygen and the ether oxygen), S304 (with the protonated nitrogen of the quinuclidine moiety) and G212 (with the hydrogen of the hydroxyl group). Only for the wild-type and CYP2D6*17 a hydrogen bonding interaction was observed between D301 and the protonated nitrogen. Hydrophobic ligand protein contacts were observed for all three cases with I106, L110, F112, F12, L121, L213, F247, L248, I297, A300, V308, and F483. The SNP F120I did not force the SoM of quinidine to move closer to the heme in order to form the 3-hydroxyquinidine metabolite as known for the F120A mutation (Fig 8F and S5 Fig) [44]. Substitution of Phe^120^ with Ile^120^ is likely to form a similar steric hindrance energy barrier considering the similarity in size and hydrophobicity of the two.

#### Bufuralol, tamoxifen and veliparib

The three substrate wild-type simulations all pointed the SoM in direction of the heme during the whole simulation time (Fig 8G–8I). Bufuralol and tamoxifen had an average SoM to heme distance of 6.0 Å and 4.4 Å respectively, whereas veliparib was kept more distant (10.4 Å) (S5 Fig). Hydrogen bonds between E216 (protonated nitrogen and the hydrogen of the hydroxyl group) and between the oxygen of the hydroxyl group and Q244, D301 and S304 were observed for bufuralol. For tamoxifen only two hydrogen bonds were observed: D301 (protonated nitrogen) and S304 (with the oxygen). Veliparib formed hydrogen bonds with G212 (hydrogens of the amine group near the oxygen), E216 (hydrogens of the protonated nitrogen), R221 (nitrogen of the imidazole), Q244 (hydrogen of the amine group near the oxygen), D301 (hydrogens of the protonated nitrogen) S304 and E215 (both with the hydrogens of the nitrogen in the imidazole). Furthermore, L110, F112, F120, L121, L213, F247, A305, V308, and F483 were the major stabilizing hydrophobic interactions observed for all three substrates.

#### Crystal structure comparison

To assess the dynamic behaviour of six key active site residues (F120, E216, Q244, D301, S304 and F483) in the presence of different ligands and among the different variants, the most prevalent conformation was aligned with the x-ray structure (4WNU or 3QM4) (S5 Fig).

The side-chain of F120 and S304 did show variation in the torsion angles. A reversed positioning of the S304 side chain was observed compared to the x-ray structure for 5 of the 15 conformations (wt_apo, V17_apo, wt_pri, wt_tam, V53_qui). E216 showed a small displacement in most conformations. Largest displacements among all conformations were found for F483 and Q244. Both residues are located at more flexible regions of the CYP2D6. As previous research indicated, F483 (located at the β 4–2 sheet and SRS 6) is known to fulfil an important role in the binding of the ligand, and also to be close to the solvent channel [10,15,56]. Experiment showed that the F483I mutant was able to metabolize testosterone, whereas wild-type was not [57]. Prinomastat (wt_pri, V53_pri) and veliparib (wt_vel) both show F483 to be moved further away compared to the x-ray position. These two ligands are considerably large and contain several bulky groups. We propose that the binding energetics (unfavourable interactions) will induce F483 to move away and the ligand size will have a determining role in the location of F483 within the active site. The flexibility and displacement of Q244 (located at the FG loop or extended G helix and SRS 3) is coupled to the flexibility of the FG loop. For instances, the wild-type simulation with bufuralol and prinomastat showed overall a more stable behaviour of the enzyme with no extreme fluctuations of the FG loop (Fig 7), which is reflected in the similar position compared to the x-ray structure. On the other hand, the wild-type with veliparib showed larger fluctuations of the FG loop which in turn led to larger displacement of Q244.

## Conclusion

This long time-scale (1 micro second) MD simulation study confirmed earlier observed CYP2D6 dynamics and provided additional insights on the structure-function relationship of the enzyme. The wild-type MD simulations showed a stabilizing effect of the ligand on the structure. The folding of primarily the FG loop and secondary the AB- and BC loop around the active site is reshaped upon the ligand binding, which contributed also to the larger identified volumes and a more closed (semi-closed) state. Differences in flexibility and arrangement of the key residues lining the active site, together with the intra- and intermolecular forces among the variants with changed enzymatic activity, suggest the need for a precise positioning of these factors to control optimal proceeding of the catalytic reaction, which is tightly coupled to the kinetics of the enzyme. The location (e.g. at the active site, or SRS) and type (conservation) of amino acid mutation appears to be relevant for maintaining a functional structural fold as well as for the regulation mechanisms (e.g. hydrogen binding network) the enzyme employs to steer the binding of ligands and cofactors. Hence, simulating the enzyme dynamics on a long time scale in the presence of explicit solvent is important for a proper understanding of the activity of the enzyme under various conditions (e.g. substrate, inhibitor, polymorphs, etc.). Such mechanistic information is of particular relevance for the drug development process as it can be directly utilized within the design of drugs in order to rationally avoid or at least limit the cytochrome liability. The observed differences among wild-type and clinically relevant allelic variants justify the need of their detailed screening using i*n silico* approaches based on docking and MD simulations.

In addition, considering the importance of the thermodynamics of the catalytic reaction, additional polymorphism studies focused on determining the free-energy barrier changes would be valuable to improve the link between differences in observed dynamical behaviour and enzyme activity. Undoubtedly, an important limitation of this study is the missing anchor part. Since CYP2D6 is anchored to the membrane at the N-terminus site, we expect less fluctuations and possibly slightly altered arrangement of the domains in the close vicinity of the membrane bilayer. We assume that this could have impact also on the tunnel structure and function. Therefore, we aim at performing additional CYP2D6 studies with protein natively anchored to the membrane.

## Supporting information

S1 Table Overview of amino acid property change by each mutation. (PDF)

S2 Table MD simulations parameter calculations for all CYP2D6 variants. (PDF)

S3 Table Overview ligand properties. (PDF)

S4 Table Replica runs of apo wild-type (2) RMSD and RMSF values compared. (PDF)

S1 Fig Box-and-whisker plots for the BC and FG loops calculated over the whole trajectory for all CYP2D6 variants. (PDF)

S2 Fig Backbone RMSD graphs for all CYP2D6 simulations–last 100 ns. (PDF)

S3 Fig Backbone RMSD graph for the apo CYP2D6 wild-type calculated over 1.4 μs. (PDF)

S4 Fig RMSD calculated over 1.0 μs for the holo CYP2D6 variants and SoM-heme distance. (TIF)

S5 Fig Key residues (E216, D301, F120, F483, S304 and Q244) located in the active site of each CYP2D6 most prevalent conformation compared to the x-ray structures. (PDF)
