# Supplementary material for: Microsecond MD simulations of human CYP2D6 wild-type and five allelic variants reveal mechanistic insights on the function
Source: PLoS One. 2018 Aug 22;13(8):e0202534. doi: 10.1371/journal.pone.0202534 (PMC6104999; doi:10.1371/journal.pone.0202534)
Supplement: S1 Table — (PDF) [file pone.0202534.s001.pdf]

Table S1. **Overview of amino acid property change by each mutation.**

CYP2D6 Mutation	Polarity	change	Hydrophobicity/hydrophilicity	change	Charge change	Lipophilic character change
**1. P34S**	nonpolar to polar	√	hydrophobic to hydrophilic	√	neutral to neutral	--
**2. L91M**	nonpolar to nonpolar	x	hydrophobic to hydrophobic	x	neutral to neutral	-
**3. H94R**	neutral/basic polar to basic polar	x	hydrophilic to hydrophilic	x	neutral to positive	none
**4. T107I**	polar to neutral nonpolar	√	hydrophilic to hydrophobic	√	neutral to neutral	++
**5. F120I**	nonpolar to nonpolar	x	hydrophobic to hydrophobic	x	neutral to neutral	+
**6. A122S**	nonpolar to polar	√	hydrophobic to hydrophilic	√	neutral to neutral	-
**7. R296C**	basic polar to nonpolar	√	hydrophilic to hydrophilic	x	positive to neutral	++
**8. S486T**	polar to polar	x	hydrophilic to hydrophilic	x	neutral to neutral	none
