# Supplementary material for: Microsecond MD simulations of human CYP2D6 wild-type and five allelic variants reveal mechanistic insights on the function
Source: PLoS One. 2018 Aug 22;13(8):e0202534. doi: 10.1371/journal.pone.0202534 (PMC6104999; doi:10.1371/journal.pone.0202534)
Supplement: S2 Table — (PDF) [file pone.0202534.s002.pdf]

Table S2. **MD simulations parameter calculations for all CYP2D6 variants.** The Solvent Accessible Surface Area (SASA) (FreeSASA v2.0.2), buried solvent area (PISA webserver) and overall volume (POVME v2.0) were calculated for the CYP2D6 dominant cluster conformation for each CYP2D6 variant.

| **#1 Cluster Conformation CYP Variant** | **Total SASA (Å$^2$)** | **% of total area** | **Polar SASA (Å$^2$)** | **% of total area** | **Apolar SASA (Å$^2$)** | **% of total area** | **BSA (Å$^2$)** | **% of total surface area** | **Volume (Å$^3$)** | **SOM Fe distance (Å)** | **ΔRMSD (vs. 4wnu)** |
|---|---|---|---|---|---|---|---|---|---|---|---|
| **wt_a** | 22195 | 93.8 | 9494 | 40.1 | 12701 | 53.7 | 1463 | 6.2 | 635 | na | 2.2 |
| **V2_a** | 21862 | 93.9 | 9248 | 39.7 | 12614 | 54.2 | 1408 | 6.1 | 716 | na | 2.6 |
| **V10_a** | 22283 | 93.7 | 9588 | 40.3 | 12694 | 53.4 | 1487 | 6.3 | 797 | na | 1.9 |
| **V17_a** | 22603 | 93.7 | 9783 | 40.6 | 12819 | 53.1 | 1522 | 6.3 | 663 | na | 2.8 |
| **V4_a** | 22072 | 94.6 | 9333 | 40.0 | 12738 | 54.6 | 1271 | 5.4 | 582 | na | 2.2 |
| **V53_a** | 24022 | 94.2 | 10132 | 39.7 | 13890 | 54.5 | 1471 | 5.8 | 836 | na | 2.8 |
| **wt_tam** | 22477 | 96.1 | 9274 | 39.7 | 13203 | 56.5 | 902 | 3.9 | 734 | 4.4 | 2.4 |
| **wt_vel** | 22398 | 94.0 | 9134 | 38.3 | 13264 | 55.7 | 1427 | 6.0 | 567 | 10.4 | 2.3 |
| **wt_buf** | 23246 | 94.6 | 10025 | 40.8 | 13221 | 53.8 | 1319 | 5.4 | 707 | 6 | 1.8 |
| **wt_pri** | 21745 | 90.3 | 8998 | 37.3 | 12748 | 52.9 | 2348 | 9.7 | 671 | 14 | 2.6 |
| **V17_pri** | 22779 | 90.1 | 9385 | 37.1 | 13394 | 53.0 | 2496 | 9.9 | 790 | 7 | 1.8 |
| **V53_pri** | 22584 | 90.2 | 9651 | 38.6 | 12933 | 51.7 | 2440 | 9.8 | 905 | 6 | 2.8 |
| **wt_qui** | 22579 | 91.5 | 9450 | 38.3 | 13128 | 53.2 | 2088 | 8.5 | 801 | 6 | 2.6 |
| **V17_qui** | 22018 | 90.5 | 9324 | 38.3 | 12694 | 52.2 | 2300 | 9.5 | 721 | 11.2 | 1.8 |
| **V53_qui** | 22600 | 91.3 | 9348 | 37.7 | 13252 | 53.5 | 2163 | 8.7 | 892 | 10.8 | 2.4 |
| **4wnu *(qui)*** | 20373 | 89.0 | 8612 | 37.6 | 11761 | 51.4 | 2509 | 11.0 | 856 | na | na |
| **2f9q *(apo)*** | 20762 | 86.0 | 8885 | 36.8 | 11878 | 49.2 | 3369 | 14.0 | 814 | na | 0.6 |
| **3qm4 *(pri)*** | 20306 | 90.6 | 8247 | 36.8 | 12060 | 53.8 | 2096 | 9.4 | 872 | na | 1.0 |
