# Supplementary material for: Microsecond MD simulations of human CYP2D6 wild-type and five allelic variants reveal mechanistic insights on the function
Source: PLoS One. 2018 Aug 22;13(8):e0202534. doi: 10.1371/journal.pone.0202534 (PMC6104999; doi:10.1371/journal.pone.0202534)
Supplement: S3 Table — (PDF) [file pone.0202534.s003.pdf]

Table S3. **Overview ligand properties.**[1]

ligand	PSA ($Å^2$)	LogP	LogS	H_{Don}	H_{Acc}	$\#Bond_{rotatable}$	Molar volume (cm^3)
**tamoxifen**	12.5	7.1	-5.6	0	2	8	356
**veliparib**	83.8	1.1	-3.0	3	3	2	192
**bufuralol**	45.4	3.2	-3.9	2	3	5	245
**prinomastat**	112.3	1.7	-4	2	6	4	307
**quinidine**	45.6	2.8	-3.0	1	4	4	266

1 Information obtained from DrugBank and Chemspider
