# Supplementary material for: Microsecond MD simulations of human CYP2D6 wild-type and five allelic variants reveal mechanistic insights on the function
Source: PLoS One. 2018 Aug 22;13(8):e0202534. doi: 10.1371/journal.pone.0202534 (PMC6104999; doi:10.1371/journal.pone.0202534)
Supplement: S4 Table — (PDF) [file pone.0202534.s004.pdf]

Table S4. **Replica runs (2) of apo wild-type, RMSD and RMSF values compared.**

simulation	mean RMSD (Å)	SD	mean RMSF (Å)	SD
**wt_a**	5.4	0.6	1.53	1.19
**wt_a_R1**	5.6	0.6	1.59	1.21
**wt_a_R2**	6.5	0.7	1.88	1.7
