# Supplementary material for: Microsecond MD simulations of human CYP2D6 wild-type and five allelic variants reveal mechanistic insights on the function
Source: PLoS One. 2018 Aug 22;13(8):e0202534. doi: 10.1371/journal.pone.0202534 (PMC6104999; doi:10.1371/journal.pone.0202534)
Supplement: S1 Fig — (PDF) [file pone.0202534.s005.pdf]

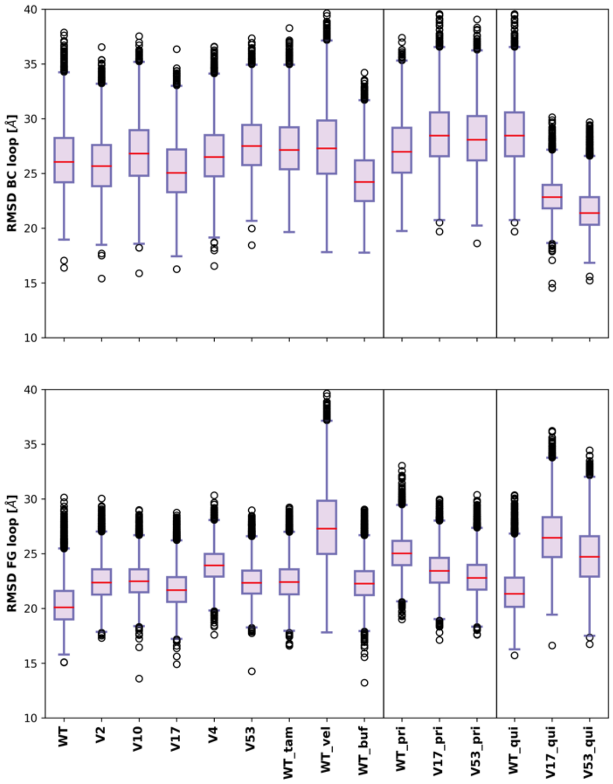

Figure S1. **Box-and-whisker plots for the BC and FG loops calculated over the whole trajectory for all CYP2D6 variants**. The distribution of the data points: the pink boxes indicate the upper - and lower quartile (25% of the data is greater than or less than this value), the caps indicate the greatest and the smallest value excluding outliners. The outliners represent more than or less than 3/2 times the upper or lower quartile, and the red line indicate the median value.
