# Supplementary material for: Microsecond MD simulations of human CYP2D6 wild-type and five allelic variants reveal mechanistic insights on the function
Source: PLoS One. 2018 Aug 22;13(8):e0202534. doi: 10.1371/journal.pone.0202534 (PMC6104999; doi:10.1371/journal.pone.0202534)
Supplement: S2 Fig — (PDF) [file pone.0202534.s006.pdf]

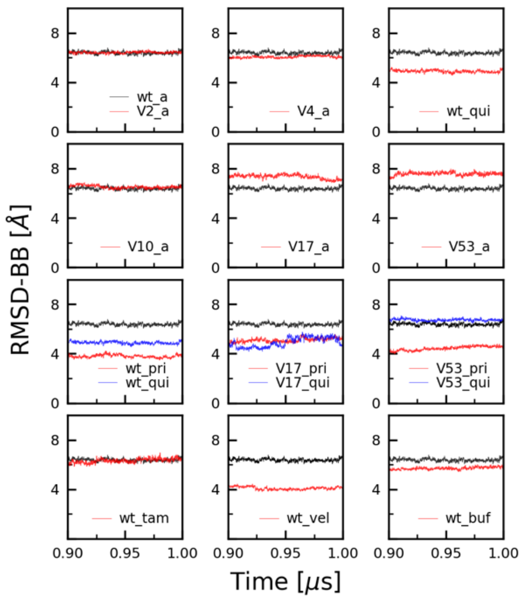

Figure S2. **Backbone root mean square deviation (RMSD) graphs for all CYP2D6 simulations – last 100 ns**. The wild-type simulation is shown black in all graphs. The RMSD graphs of the last 100 ns that confirm that the simulations were equilibrated.
