# Supplementary material for: Microsecond MD simulations of human CYP2D6 wild-type and five allelic variants reveal mechanistic insights on the function
Source: PLoS One. 2018 Aug 22;13(8):e0202534. doi: 10.1371/journal.pone.0202534 (PMC6104999; doi:10.1371/journal.pone.0202534)
Supplement: S3 Fig — (PDF) [file pone.0202534.s007.pdf]

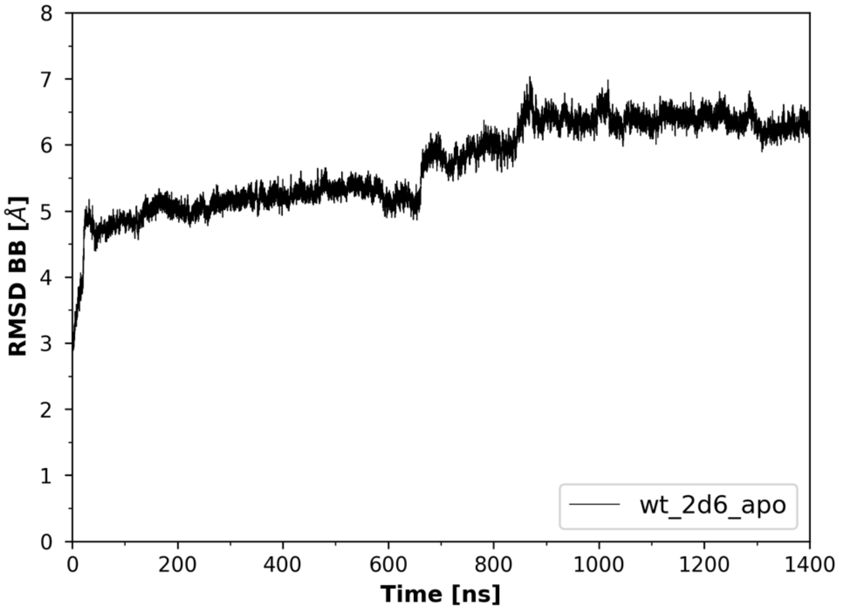

Figure S3. **Backbone root mean square deviation (RMSD) graph for the apo CYP2D6 wild-type calculated over 1.4 µs.** The additional 400 ns simulation ran for the wild-type simulation (black) confirms that the simulation reached plateau at the end.
