# Supplementary figures and images for: Microsecond MD simulations of human CYP2D6 wild-type and five allelic variants reveal mechanistic insights on the function
Source: PLoS One. 2018 Aug 22;13(8):e0202534. doi: 10.1371/journal.pone.0202534 (PMC6104999; doi:10.1371/journal.pone.0202534)

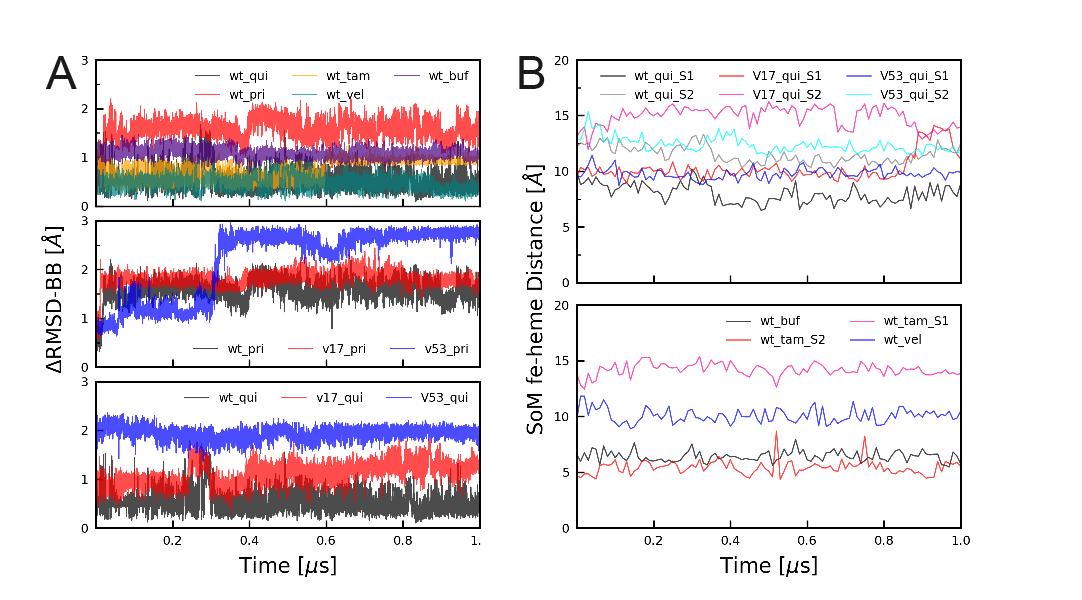

Supplement: S4 Fig — (TIF) [file pone.0202534.s008.tif]
