# Supplementary material for: Microsecond MD simulations of human CYP2D6 wild-type and five allelic variants reveal mechanistic insights on the function
Source: PLoS One. 2018 Aug 22;13(8):e0202534. doi: 10.1371/journal.pone.0202534 (PMC6104999; doi:10.1371/journal.pone.0202534)
Supplement: S5 Fig — (PDF) [file pone.0202534.s009.pdf]

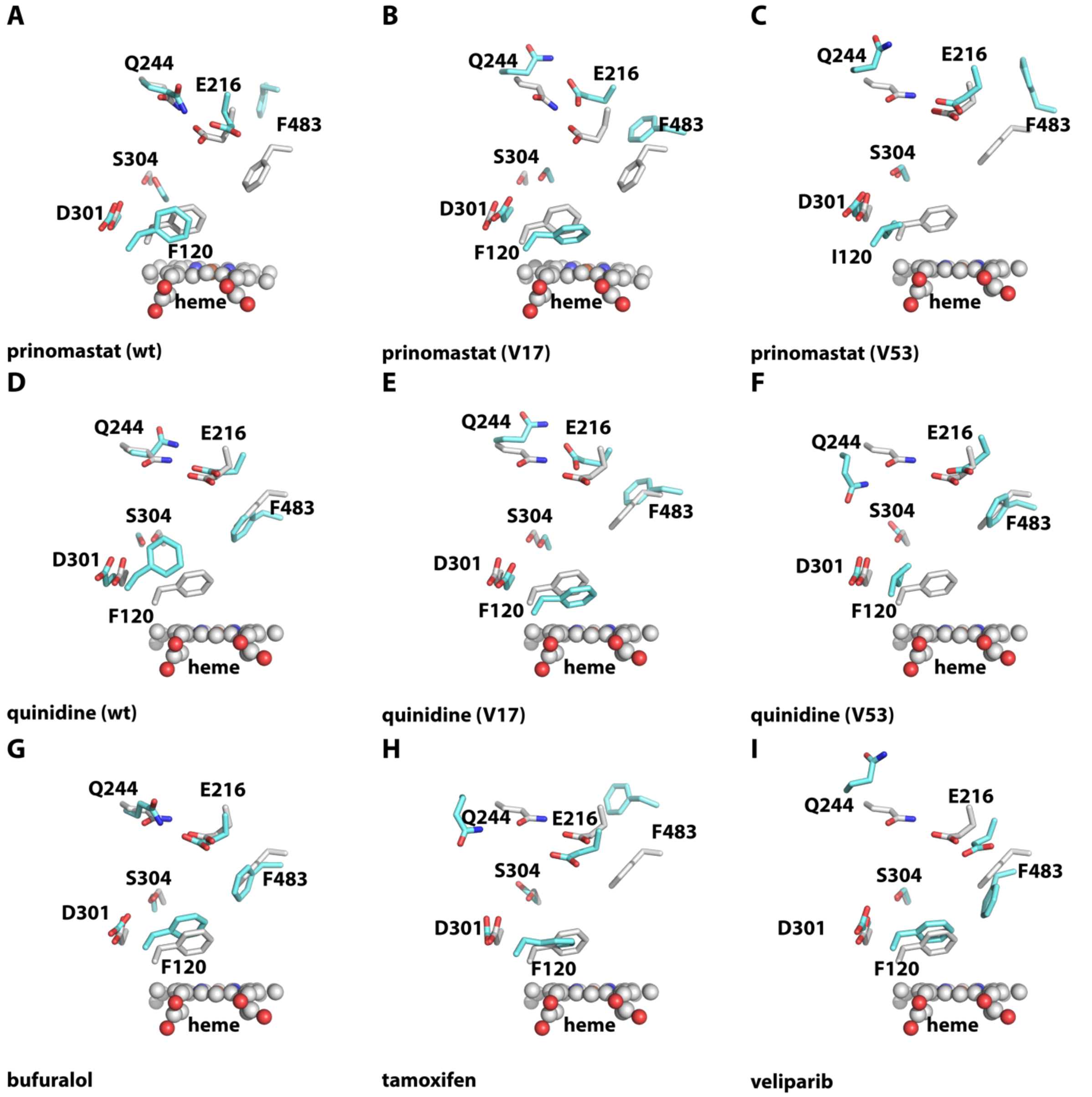

Figure S5. **Key residues (E216, D301, F120, F483, S304 and Q244) located in the active site of each CYP2D6 most prevalent conformation compared to the x-ray structures (ligands are left out for clarity).** The active site is shown for the most prevalent conformation in cyan, and the x-ray structure in white (A-C: 3QM4, D-I: 4WNU). **(A-C)** Prinomastat (inhibitor) with wild-type (wt) (A), CYP2D6*17 (V17) (B) and CYP2D6*53 (V53) (C), **(D-F**) Quinidine (inhibitor) with wild-type (wt) (D), CYP2D6*17 (V17) (E) and CYP2D6*53 (V53) (F), **(G-I)** wild-type together with substrates: bufuralol (G), tamoxifen (H), and veliparib (I).
